# Supplementary material for: Evolution of the ionisation energy with the stepwise growth of chiral clusters of [4]helicene
Source: Nat Commun. 2024 Jun 10;15:4928. doi: 10.1038/s41467-024-48778-0 (PMC11164862; doi:10.1038/s41467-024-48778-0)
Supplement: Supplementary file 1 — REVISED Supplementary Information [file 41467_2024_48778_MOESM1_ESM.pdf]

Supplementary Information

Evolution of the ionisation energy with the stepwise growth of chiral clusters of [4]helicene

S. R. Domingos,[†,‡] D. S. Tikhonov,[†] A. L. Steber,[†,¶] P. Eschenbach,[§,‖] S. Gruet,[†] H. R. Hrodmarsson,[⊥,#] K. Martin,[@] G. A. Garcia,[⊥] L. Nahon,[⊥] J. Neugebauer,[§,‖] N. Avarvari,[@] and M. Schnell[*,†,△]

†*Deutsches Elektronen-Synchrotron DESY, Notkestr. 85, 22607 Hamburg, Germany*

‡*Present address: CFisUC, Department of Physics, University of Coimbra, 3004-516 Coimbra, Portugal*

¶*Present address: Department of Physical Chemistry, Faculty of Science, University of Valladolid, 47011 Valladolid, Spain*

§*Organisch-Chemisches Institut, University of Münster, D-48149 Münster, Germany*

‖*Center for Multiscale Theory and Computation (CMTC), University of Münster, D-48149 Münster, Germany*

⊥*Synchrotron SOLEIL, L'Orme des Merisiers, 91192 Gif sur Yvette Cedex, France*

#*Present address: LISA UMR 7583 Université Paris-Est Créteil and Université de Paris, Institut Pierre et Simon Laplace, 61 Avenue du Général de Gaulle, 94010 Créteil, France*

@*Univ Angers, CNRS, MOLTECH-Anjou, SFR MATRIX, F-49000 Angers, France*

△*Institute of Physical Chemistry, Christians-Albrecht-Universität zu Kiel, Kiel, Germany*

E-mail:

sergio.domingos@uc.pt,denis.tikhonov@desy.de,amanda.steber@uva.es,melanie.schnell@desy.de

Contents

Computational Details **3**

Structure Setup and Geometry Optimization 3

Calculation of Vertical Ionization Potentials 3

Theoretical ionization potential of the [4]helicene clusters **8**

Fit of the first peaks in PEPICO spectra of the [4]helicene monomer **19**

Supplementary Computational Details

Photoelectron spectra of the [4]helicene

Calculations of the vibrationally-resolved photoelectron spectra (PES) of the [4]helicene molecule were done at the PBE0[1]/def2-TZVPP[2] level of theory. The geometries of the [4]helicene and of its cation in the ground and five excited states were optimized, and the Hessians at each equilibrium structure were computed. The resulting spectrum was computed using the ezFCF (formerly ezSpectrum) program.[3] In addition to that, the vertical ionization potentials were computed at the geometry of the neutral [4]helicene.

Structure Setup and Geometry Optimization

The structures given in this work were obtained by the following procedure.

1. The initial structures were obtained by:
   - global optimization using the ABCluster program;[4–6]
   - generation of trial cluster geometries via Coalescence-Kick software.[7,8]
2. The resulting guess structures were optimized at the GFN2-xTB level of theory[9] using the XTB software.[10]
3. Only the structures with relative energy smaller than 300 K (210 cm^{-1}) were kept for each cluster size/type (i.e., for each n and m in cluster $\mathrm{P_nM_m}$).
4. All the structures were adjusted to have the center of mass as the coordinates origin.
5. The distance matrix $\mathcal{D}$ (consisting of elements $D_{\alpha\beta}$) between the kept structures was calculated by minimizing the functional

$$\Phi_{\alpha\beta}(\phi,\theta,\chi)=\frac{\sum_{i=1}^{N}\sum_{j=1}^{N}(r_{ij}^{\alpha\beta})^2\cdot w_{ij}^{\alpha\beta}}{\sum_{i=1}^{N}\sum_{j=1}^{N}w_{ij}^{\alpha\beta}}\ . \tag{1}$$

The $r_{ij}^{\alpha\beta} = |\mathbf{r}_i^\alpha - \mathcal{R}(\phi, \theta, \chi)\mathbf{r}_j^\beta|$ is the distance between the coordinates of the atom $\#i$ of structure α and atom $\#j$ of structure β, that was rotated via rotation matrix $\mathcal{R}$ dependent on three Euler angles (ϕ, θ, χ). Weights $w_{ij}^{\alpha\beta}$ are calculated via equation:

$$w_{ij}^{\alpha\beta} = \begin{cases} 0 \text{ if atoms } i \text{ and } j \text{ are of the different type,} \\ \exp\left(-\frac{(r_{ij}^{\alpha\beta})^2}{(s \cdot R_i)^2}\right) \end{cases} \tag{2}$$

here R_i denotes atomic radius of the atom i, which was taken 0.53 Å for the hydrogens, and 1.20 Å for carbons. Scale coefficient s was taken to be 50. Atoms i and j were considered as the same type if:

- they had the same nuclei charge,
- they had the same number of chemically-bonded neighbours,
- the masses of their chemically-bonded neighbours were the same.

The distance $D_{\alpha\beta}$ between α and β was considered to be $D_{\alpha\beta} = \min(\Phi_{\alpha\beta}(\phi, \theta, \chi))$. Minimzation was performed using differential evolution algorithm as implemented in the SciPy library.

6. The structures α and β were considered to be the same, if the following criterion was fulfilled:

$$\left| \frac{|D_{\alpha\beta}|}{\sqrt{D_{\alpha\alpha} \cdot D_{\alpha\beta}}} - 1 \right| < \max(D_{\alpha'\beta'}) \cdot \theta \ , \tag{3}$$

where θ is the threshold value taken to be 0.05, and $\max(D_{\alpha'\beta'})$ is the maximal value of the distance matrix $\mathcal{D}$.

7. Amongst the same structural isomers, the lowest energy ones are taken.

Steps 2-6 above are performed via a script `findUniqueConformers.py` of the Molinc project.[11]

Calculation of Vertical Ionization Potentials

Vertical ionization potentials (vIP) for all helicene complexes were calculated using i) DLPNO-CCSD(T),[12–14] ii) IP-EOM-DLPNO-CCSD[15–17] and iii) a combination of diabatic ionization potentials obtained with subsystem-based G_0W_0[18] and electronic couplings from FDE-ET.[19–22] In case of DLPNO-CCSD(T), the total energies of the neutral and cationic complexes were calculated, where the energy difference is the vIP. In case of IP-EOM-DLPNO-CCSD the vIPs were directly obtained from the calculation. Both types of calculations were performed using the ORCA[23,24] program package. For all calculations the cc-pVTZ[25] basis set and `NormalPNO` thresholds were used. To reduce computational cost, the resolution-of-the-identity (RI) approximation in conjunction with the auxiliary Coulomb fitting bases cc-pVTZ/C[26] and Def2/J[27,28] was employed. Additionally, the chain-of-spheres exchange (COSX) algorithm[29] was applied to numerically fit the exchange integrals to further reduce the computational cost. For the combination of diabatic ionization potentials obtained with subsystem-based G_0W_0 and electronic couplings from FDE-ET a locally modified version of the SERENITY[30,31] program was used. All calculations were carried out using the cc-pVTZ[25] basis set. To reduce computational cost, the RI approximation in conjunction with the auxiliary Coulomb fitting basis Def2/J[27,28] was employed. For the FDE-ET and G_0W_0 calculations the PBE[32] and BHLYP[33,34] exchange–correlation (XC) functionals were used, respectively. For the non-additive contributions the PW91[35,36] XC functional was used in combination with the conjoint[37] kinetic-energy functional PW91k.[38] Mutual relaxations of subsystem densities were accounted for using Freeze-and-Thaw (FaT) cycles.[39] Three such cycles were found sufficient to obtain accurate electronic densities (see also Refs. 40–42). The FDE-ET electronic couplings V_{ij} were calculated by coupling two quasi-diabatic states Φ_i and Φ_j, where helicene monomer i or j is charged, including all subsystems contained in a cluster. To give an example, to calculate V_{12} of the complex dimer the states $\Phi_1 = |[^1\text{hel}^{\cdot+}{-}^2\text{hel}]\rangle$ and $\Phi_2 = |[^1\text{hel}_1{-}^2\text{hel}^{\cdot+}]\rangle$ are constructed and coupled. In case of e.g. the tetramer V_{12} is calculated from coupling $\Phi_1 = |[^1\text{hel}^{\cdot+}{-}^2\text{hel}{-}^3\text{hel}{-}^4\text{hel}]\rangle$ and $\Phi_2 = |[^1\text{hel}{-}^2\text{hel}^{\cdot+}{-}^3\text{hel}{-}^4\text{hel}]\rangle$.

Here, ^{i}hel corresponds to one of the helicene monomers. For obtaining diabatic ionization potentials H_{ii}, G_0W_0 calculations were carried out including the energetically highest occupied and lowest virtual orbitals. Furthermore, 60 integration points, obtained from a modified Gauss-Legendre quadrature, along the imaginary frequency axes have been employed throughout. The analytic continuation approach[43] was employed using 16 Padé points.[44,45] For each complex electronic couplings V_{ij} and diabatic diabatic ionization potentials H_{ii} were obtained and assembled in a matrix $\mathbf{M}$ whose elements are defined by $M_{ij} = V_{ij}$ for $i \neq j$ and $M_{ij} = H_{ii}$ for $i = j$. This matrix is diagonalized and a set of eigenvalues is obtained. The lowest eigenvalue provides the lowest vIP for each helicene complex.

In addition to the fully *ab initio* methods, we have also applied the two semi-empirical methods of calculating the vIPs:

- DFTB-CI,[46,47] from the deMonNano package,[48]
- IP-xTB2 model from the XTB package.[10]

Racemization barrier of [4]helicene monomer and dimer

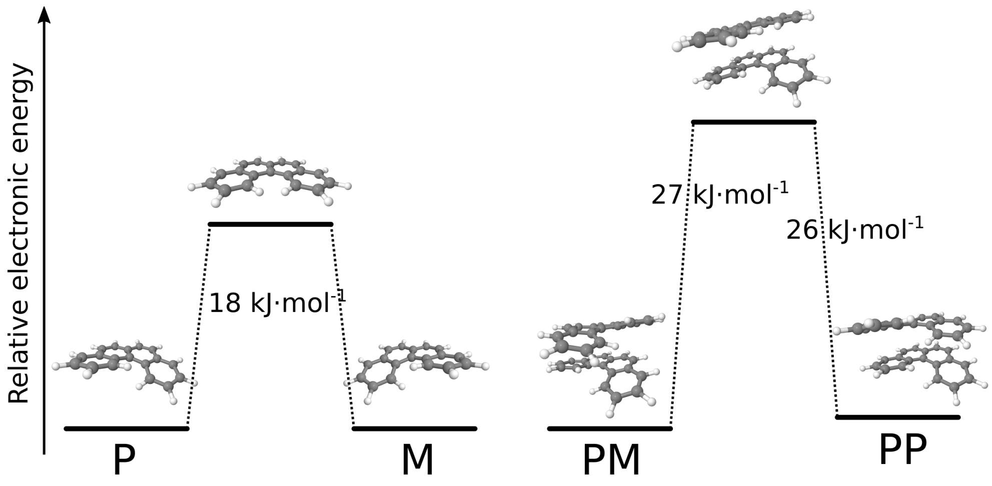

Supplementary Figure 1: **Racemization barriers.** Comparison of calculated activation energies for the racemization of the [4]helicene monomer and dimer.

The equilibrium structure (ES) and a planar transition state (TS) of the neutral [4]he-

licene were optimized at the PBE0-D3(BJ)/def2-TZVPP level of theory. For both the optimized structures at DLPNO(TightPNO)-CCSD(T)/cc-pVTZ, single-point energies were computed. The electronic energy difference between the TS and ES was found to be 0.19 eV (18 kJ mol^{-1}) at both PBE0-D3(BJ)/def2-TZVPP and DLPNO(TightPNO)-CCSD(T)/cc-pVTZ//PBE0-D3(BJ)/def2-TZVPP levels of theory. The zero-point-energy-corrected barrier height is 0.18 eV, and the Gibbs free energy barrier at 298 K is 0.19 eV.

A similar calculation for the interconversion barrier for PP$\leftrightharpoons$PM was performed. The lowest energy PP and PM clusters of [4]helicene at GFN2-xTB level of theory were reoptimized at the PBE0-D3(BJ)/def2-SV(P) level of theory. The harmonic frequency calculation confirmed the ES structures at the same level of theory. The basis set was reduced from def2-TZVPP to def2-SV(P) to deal with the increasing cost of the calculation in the case of the TS search. For the two optimized ES of PP and PM, a climbing image nudged elastic band method (CI-NEB) TS search[49] and consecutive harmonic frequency calculation were performed at the PBE0-D3(BJ)/def2-SV(P) level of theory. The energies of the two ES and of the TS were recomputed at the DLPNO(NormalPNO)-CCSD(T)/cc-pVTZ//PBE0-D3(BJ)/def2-SV(P) level of theory. The electronic energy differences between the TS and ES were found to be

- 0.23 eV (23 kJ mol^{-1}) for conversion PM$\rightarrow$PP and 0.22 eV (21 kJ mol^{-1}) for conversion PP$\rightarrow$PM at the PBE0-D3(BJ)/def2-SV(P) level of theory;
- 0.28 eV (27 kJ mol^{-1}) for conversion PM$\rightarrow$PP and 0.27 eV (26 kJ mol^{-1}) for conversion PP$\rightarrow$PM at the DLPNO(NormalPNO)-CCSD(T)/cc-pVTZ//PBE0-D3(BJ)/def2-SV(P) level of theory.

Here, the difference between DFT and DLPNO-CCSD(T) energies is more noticeable, probably because of the decrease in the basis set for DFT, but also probably due to the higher role of the dispersion interaction in the dimer. Thus, the ZPVE-corrected values and Gibbs free energy barrier were only computed at the DLPNO(NormalPNO)-CCSD(T)/cc-pVTZ//PBE0-

D3(BJ) level of theory. ZPVE-corrected barriers are 0.28 eV for conversion PM→PP and 0.26 eV for conversion PP→PM. Gibbs free energy barriers at 298 K are 0.31 eV for conversion PM→PP and 0.29 eV (for conversion PP→PM).

The comparison of the relative electronic energies at the DLPNO-CCSD(T)/cc-pVTZ//DFT level of theory for P↔M and PM↔PP interconversions, as well as ES and TS structures are given in Figure 1. All the calculations were performed using ORCA software.

Theoretical ionization potentials of the [4]helicene clusters

In the main text, a lowest-energy structure set A according to the GFN2-xTB electronic energies has been defined. This has been done for reasons of consistency with the geometry optimization of these structures that has been carried out using the same method. In this supplementary material, we will consider an additional lowest-energy structure set B according to electronic energies obtained with DLPNO-CCSD(T) (see Tab. 6). The [4]helicene cluster geometries are given in the previous section. Calculated vIPs are shown in Tab. 2. The vIPs calculated using DLPNO-CCSD(T), IP-EOM-DLPNO-CCSD and G_0W_0–FDE-ET are shown in Fig. 2. As can be seen the vIPs calculated with DLPNO-CCSD(T) and G_0W_0–FDE-ET are very close to the experimentally obtained ionization thresholds (IT) for nearly each [4]helicene cluster. The deviation from the experimental ITs increases for the cluster sizes 6 and 7. Fig. 2 indicates that for those cluster sizes, there are conformers with a vIP perfectly reproducing the experimental IT. However, those conformers do not belong to the earlier mentioned structure set B with the lowest electronic ground-state energy according to DLPNO-CCSD(T) (see Tab. 6 and Fig. 3). For smaller cluster sizes the overall trend of the ITs is very well reproduced. The same holds for the vIPs calculated with IP-EOM-DLPNO-CCSD. In contrast to DLPNO-CCSD(T) or G_0W_0–FDE-ET the vIPs are shifted to smaller values and, thus, underestimate the experimental ITs. Fig. 4 shows the kernel-density estimation[50,51] (KDE) curves and root-mean square deviation (RMSD) of the

difference of the DLPNO-CCSD(T), IP-EOM-DLPNO-CCSD and G_0W_0–FDE-ET vIPs with respect to (w.r.t.) the experimental ionization thresholds calculated using the SEABORN[52,53] Python library. The difference of the theoretical and experimental values where calculated for every [4]helicene cluster and the probability density function (PDF) was calculated using the KDE method. When comparing the KDE curves it can be seen that the curve of G_0W_0–FDE-ET is the most narrow, while IP-EOM-DLPNO-CCSD shows a wide distribution of possible vIPs. Additionally, the maximum of the G_0W_0–FDE-ET KDE curve is the closest to 0 eV, which indicates that this method provides the most accurate results beneath the three chosen approaches.

Supplementary Table 1: **Vertical and adiabatic ionization potentials.** (vIP and aIP) of [4]helicene were computed using PBE0/def2-TZVPP and TD-DFT-PBE0/def2-TZVPP. All values are given in units of eV.

[4]helicene$^+$ state number	vIP	aIP
0	7.4	7.3
1	7.9	7.8
2	9.0	8.9
3	10.0	9.0
4	10.2	9.8
5	10.5	10.1

Supplementary Table 2: **Vertical ionization potentials of the lowest energy [4]helicene clusters (vIP) and relative energies of each cluster geometry with respect to the lowest structure of the same size (ΔE).** The geometries and energies (ΔE) were obtained with GFN2-xTB. The vIPs were computed using DLPNO-CCSD(T), IP-EOM-DLPNO-CCSD, G_0W_0–FDE-ET, DFTB-CI, and IP-xTB2. The vIPs are given in units of eV, ΔE values are given in units of cm^{-1}. # indicates the isomer number with respect to the most stable (#=0) for each cluster type.

Cluster	#	ΔE	vIP				
			DLPNO-CCSD(T)	IP-EOM-DLPNO-CCSD	G_0W_0–FDE-ET	DFTB-CI	IP-xTB2
			Monomer				
P	0	0	7.679	7.483	7.499	7.6072	7.5801
			Dimers				
PM	0	301	7.309	7.020	7.210	7.3938	7.2315
PM	1	310				7.3993	7.2252
PM	2	339				7.4000	7.2440
PM	3	344				7.3931	7.2168
PM	4	353				7.4039	7.2350
PM	5	369				7.3948	7.2284
PM	6	374				7.3935	7.2180
PM	7	386				7.3922	7.2279
PM	8	398				7.4049	7.2134
PP	0	0	7.249	6.916	7.147	7.3704	7.2297
PP	1	47				7.3710	7.2023
PP	2	59				7.3668	7.2475
PP	3	77				7.3725	7.1955
PP	4	78				7.3668	7.2212
PP	5	87				7.3696	7.2421
PP	6	93				7.3634	7.1907
PP	7	111				7.3647	7.1891
			Trimers				
PPM	0	281	7.083	6.746	7.061	7.3115	7.0494
PPM	1	393				7.3140	7.0121
PPM	2	421				7.3123	7.0181
PPP	0	0	6.980	6.588	6.930	7.3065	7.0204
PPP	1	22				7.3081	7.0128
PPP	2	64				7.1414	7.0573
			Tetramers				
PPMM	0	362	6.943	6.602	6.926	7.0990	6.8977
PPMM	1	445				7.2847	6.8925
PPMM	2	550				7.1182	6.9215
PPPM	0	387	not converged	6.647	6.955	7.0989	6.9086

PPPP	0	0	6.905	6.489	6.864	7.0759	6.8888
PPPP	1	45				7.2862	6.9079
Pentamers							
PPPMM	0	53	6.877	6.517	6.883	7.0757	6.8146
PPPPM	0	53	6.822	6.473	6.891	7.0223	6.8102
PPPPM	1	116				7.0503	6.8033
PPPPP	0	0	6.979	6.623	6.968	7.2765	6.7914
PPPPP	1	141				7.0612	6.8041
Hexamers							
PPPMMM	0	430	6.913	6.792	7.066	7.2881	6.8436
PPPPMM	0	529	6.670	6.367	6.778	7.3967	6.7839
PPPPMM	1	608				7.1253	6.7986
PPPPPM	0	508	6.802	6.640	6.554	7.3235	6.8225
PPPPPM	1	611				7.1551	6.8155
PPPPPP	0	0	7.164	6.536	6.890	7.3186	6.8282
Heptamers							
PPPPMMM	0	911	not converged	6.731	7.007	7.2336	6.8069
PPPPMMM	1	923				7.2765	6.7474
PPPPMMM	2	978				7.0403	6.6323
PPPPMMM	3	1095				6.9637	6.7610
PPPPPMM	0	0	7.126	6.579	6.648	7.1767	6.7262
PPPPPPM	0	208	not converged	6.394	6.820	7.1892	6.7624
PPPPPPP	0	343	6.765	6.597	6.853	7.3633	6.7697

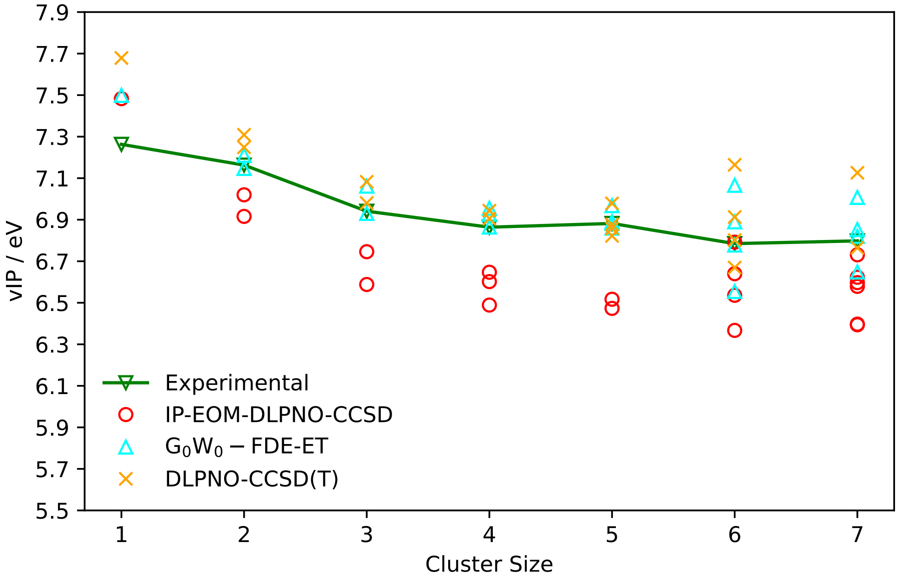

Supplementary Figure 2: **Vertical ionisation potentials vs. cluster size.** Graphical representation of calculated vertical ionization potentials vIP (in units of eV) for different helicene cluster sizes using different methods. Each point represents one distinct conformer. Additionally, experimentally obtained ionization thresholds are shown.

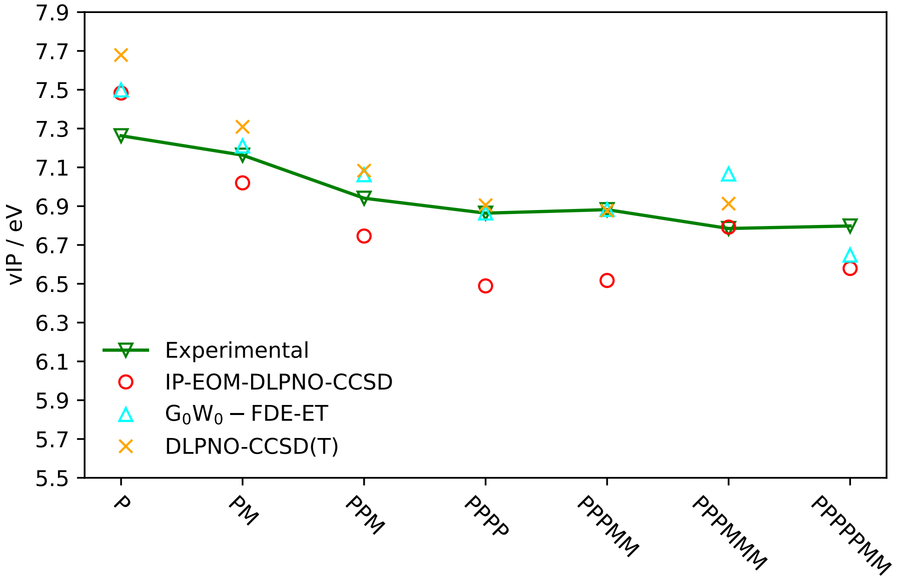

Supplementary Figure 3: **Vertical ionisation potentials for the lowest energy clusters.** Graphical representation of calculated vIPs (in units of eV) for the lowest energy helicene clusters (according to DLPNO-CCSD(T) in Tab. 6) using different methods. Additionally, experimentally obtained ionization thresholds are shown.

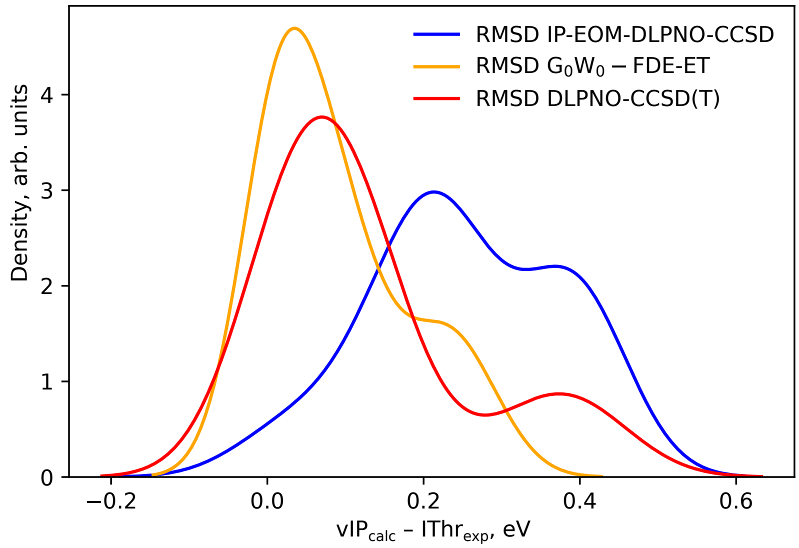

Supplementary Figure 4: **Kernel-density estimation curves** of the difference of the calculated vIPs (in units of eV) with respect to the experimental ionization thresholds.

Supplementary Table 3: **Diabatic ionization energies** H_{ii} (in units of eV) calculated for each [4]helicene cluster using the subsystem-based G_0W_0 approach.

Cluster	$H_{1,1}$	$H_{2,2}$	$H_{3,3}$	$H_{4,4}$	$H_{5,5}$	$H_{6,6}$	$H_{7,7}$
PM	7.299	7.317	–	–	–	–	–
PP	7.277	7.277	–	–	–	–	–
PPM	7.216	7.156	7.244	–	–	–	–
PPP	7.209	7.210	7.134	–	–	–	–
PPMM	7.093	7.176	7.112	7.181	–	–	–
PPPM	7.096	7.085	7.179	7.224	–	–	–
PPPP	7.075	7.175	7.177	7.070	–	–	–
PPPMM	7.066	7.165	7.192	7.070	7.088	–	–
PPPPM	7.029	7.076	7.063	7.187	7.200	–	–
PPPPP_a	7.178	7.120	7.085	7.072	7.194	–	–
PPPPP_b	7.185	7.222	7.286	7.070	7.303	–	–
PPPMMM	7.340	7.268	7.073	7.260	7.237	7.272	–
PPPPMM	7.525	7.027	7.063	6.934	7.214	7.425	–
PPPPPM	7.329	7.254	7.110	7.080	7.091	7.442	–
PPPPPP	7.375	7.346	7.195	7.207	7.023	7.036	–
PPPPMMM	7.351	7.179	7.028	7.113	7.335	7.360	7.259
PPPPPMM	7.076	7.199	6.917	7.264	7.274	7.419	7.031
PPPPPPM	7.262	7.060	7.346	7.141	7.125	7.253	7.307
PPPPPPP	7.392	7.257	7.280	7.232	7.258	7.164	7.215

Supplementary Table 4: **Frozen-Density Embedding – Electron Transfer (FDE-ET)** electronic couplings V_{ij} (in units of eV) calculated for quasi-diabatic states Φ_i and Φ_j of each [4]helicene cluster. [Part 1]

Cluster	V_{12}	V_{13}	V_{14}	V_{15}	V_{16}	V_{17}	V_{23}	V_{24}	V_{25}	V_{26}
PM	−0.098	–	–	–	–	–	–	–	–	–
PP	−0.130	–	–	–	–	–	–	–	–	–
PPM	−0.116	0.005	–	–	–	–	0.033	–	–	–
PPP	0.005	−0.206	–	–	–	–	−0.123	–	–	–
PPMM	−0.163	−0.094	0.001	–	–	–	0.003	0.000	–	–
PPPM	−0.095	0.004	−0.117	–	–	–	−0.086	0.003	–	–
PPPP	0.004	−0.124	−0.106	–	–	–	0.000	−0.208	–	–
PPPMM	−0.115	0.000	−0.113	−0.009	–	–	0.000	0.002	0.000	–
PPPPM	−0.107	−0.106	0.008	0.002	–	–	0.002	0.000	−0.033	–
PPPPP_a	0.000	0.003	−0.131	0.000	–	–	−0.163	0.007	−0.097	–
PPPPP_b	0.012	−0.017	−0.080	0.003	–	–	0.004	−0.116	−0.175	–
PPPMMM	−0.029	0.002	0.003	−0.017	0.002	–	0.000	−0.049	0.001	0.006
PPPPMM	−0.012	0.006	−0.044	−0.018	−0.077	–	0.003	−0.192	−0.022	0.001
PPPPPM	−0.009	−0.026	0.002	0.001	−0.010	–	0.610	0.005	0.000	0.000
PPPPPP	−0.150	0.003	−0.007	−0.016	−0.006	–	0.001	0.005	0.001	−0.003
PPPPMMM	0.003	−0.003	0.002	−0.013	0.005	−0.021	−0.053	0.006	−0.011	−0.020
PPPPPMM	0.000	0.337	0.001	0.000	−0.002	0.001	0.002	−0.014	0.000	−0.025
PPPPPPM	0.003	0.158	0.007	0.001	0.001	0.048	0.000	−0.006	−0.146	0.000
PPPPPPP	0.373	0.000	0.003	−0.014	−0.011	−0.018	0.007	−0.230	0.001	0.000

Supplementary Table 5: **Frozen-Density Embedding – Electron Transfer (FDE-ET)** electronic couplings V_{ij} (in units of eV) calculated for quasi-diabatic states Φ_i and Φ_j of each [4]helicene cluster. [Part 2]

Cluster	V_{27}	V_{34}	V_{35}	V_{36}	V_{37}	V_{45}	V_{46}	V_{47}	V_{56}	V_{57}	V_{67}
PM	–	–	–	–	–	–	–	–	–	–	–
PP	–	–	–	–	–	–	–	–	–	–	–
PPM	–	–	–	–	–	–	–	–	–	–	–
PPP	–	–	–	–	–	–	–	–	–	–	–
PPMM	–	−0.111	–	–	–	–	–	–	–	–	–
PPPM	–	0.000	–	–	–	–	–	–	–	–	–
PPPP	–	0.003	–	–	–	–	–	–	–	–	–
PPPMM	–	0.006	−0.028	–	–	−0.132	–	–	–	–	–
PPPPM	–	−0.102	0.000	–	–	0.000	–	–	–	–	–
PPPPP_a	–	−0.139	0.004	–	–	0.000	–	–	–	–	–
PPPPP_b	–	0.007	0.004	–	–	0.005	–	–	–	–	–
PPPMMM	–	0.001	−0.034	0.013	–	−0.003	−0.005	–	0.004	–	–
PPPPMM	–	−0.033	0.000	0.000	–	0.003	0.001	–	−0.023	–	–
PPPPPM	–	0.110	0.006	−0.010	–	−0.096	−0.025	–	0.006	–	–
PPPPPP	0.000	0.000	0.004	−0.072	0.000	−0.011	0.006	0.000	−0.131	–	–
PPPPMMM	0.000	−0.011	−0.016	−0.020	0.001	0.002	−0.011	0.009	0.000	0.008	−0.005
PPPPPMM	0.079	0.000	0.000	−0.040	−0.024	−0.144	−0.084	0.000	0.001	0.000	0.012
PPPPPPM	0.130	0.002	0.001	−0.026	0.002	−0.246	−0.039	0.001	0.004	0.008	0.000
PPPPPPP	0.004	0.006	0.003	−0.003	−0.005	0.002	0.000	0.001	−0.072	0.002	−0.221

Supplementary Table 6: **DLPNO-CCSD(T) total energies:** $E_{\mathrm{tot}}^{\text{DLPNO-CCSD(T)}}$ (in units of eV) for different [4]helicene cluster conformers.

Cluster	$E_{\mathrm{tot}}^{\text{DLPNO-CCSD(T)}}$
Heptamers	
PPPPPMM	−131786.930
PPPPPPM	−131786.865
PPPPMMM	−131786.817
PPPPPPP	−131786.675
Hexamers	
PPPMMM	−112960.193
PPPPPP	−112960.114
PPPPMM	−112960.052
PPPPPM	−112960.013
Pentamers	
PPPMM	−94133.326
PPPPP_b	−94133.276
PPPPM	−94133.261
PPPPP_a	−94133.248
Tetramers	
PPPP	−75306.520
PPMM	−75306.472
PPPM	−75306.460
Trimers	
PPM	−56479.713
PPP	−56479.669
Dimers	
PM	−37652.894
PP	−37652.870
Monomer	
P	−18826.089

Supplementary Table 7: **Dissociation energies (D_e, in eV) for the evaporation of a monomer from the neutral and cation clusters.** The neutral values have been extracted using the energies for the most stable structures in Table 6, while the ionisation energies needed to calculate the cationic values have been taken from Table 2, using the G_0W_0−FDE-ET method which is in good agreement with the experimental results.

n	$D_e(M_n \rightarrow M_{n-1} + M)$	$D_e(M_n^+ \rightarrow M_{n-1}^+ + M)$
2	0.69	1.04
3	0.74	0.96
4	0.67	0.74
5	0.70	0.60
6	0.68	0.76
7	0.57	0.81

Experimental ionization thresholds of the [4]helicene clusters

The ionization thresholds were determined as following.

- The low-energy edge of the PEPICO spectrum for each cluster size was fitted with a function

$$I(\nu) = A + B \cdot \left(1 + \mathrm{erf}\left(\frac{\nu - \nu_0}{\delta\nu}\right)\right) \ , \tag{4}$$

  where the ν — is the photon frequency, A is the baseline intensity, B is the increase height, $\mathrm{erf}(x) = \frac{2}{\sqrt{\pi}} \int_0^x \exp(-t^2) dt$ is the error function, ν_0 is the rising edge inflection point, and $\delta\nu$ is the increase width parameter.

- The ionization threshold was determined as the intersection of the tangent line at the inflection point with the baseline. This point can be expressed through the fitted parameters (A, B, ν_0, and $\delta\nu$) as

$$\nu_{\mathrm{IThr}} = \nu_0 - \frac{\sqrt{\pi}}{2} \cdot \delta\nu \tag{5}$$

  with formal uncertainty of

$$\pm \sqrt{\sigma_{\nu_0}^2 + \frac{\pi}{4} \cdot \sigma_{\delta\nu}^2} \ , \tag{6}$$

  where σ_{ν_0} and $\sigma_{\delta\nu}$ are the formal least-squares uncertainties of the ν_0 and $\delta\nu$ parameters.

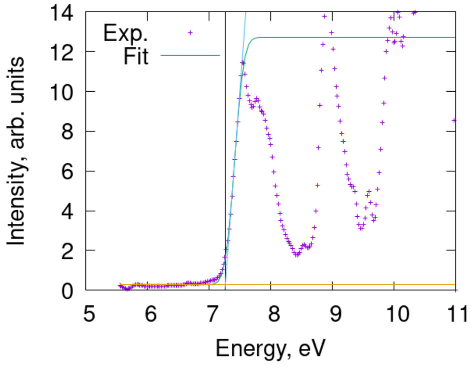

(a) $(\mathrm{Hel})_1$

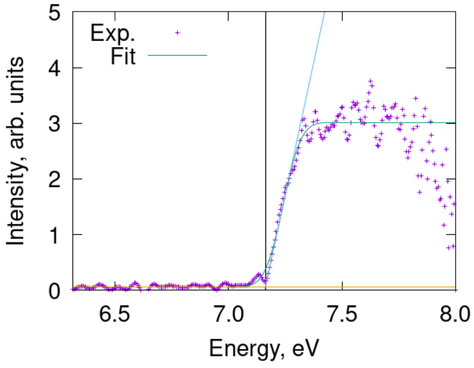

(b) $(\mathrm{Hel})_2$

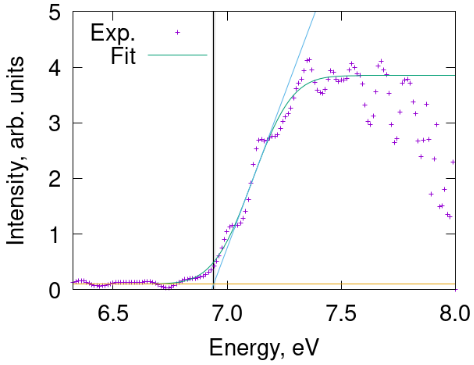

(c) $(\mathrm{Hel})_3$

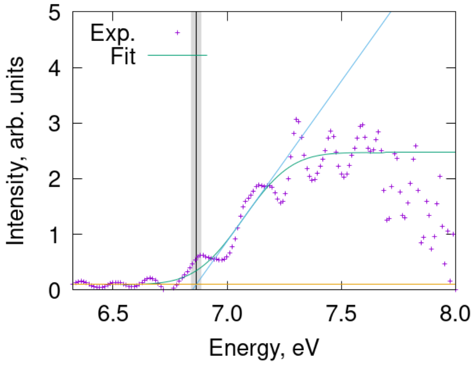

(d) $(\mathrm{Hel})_4$

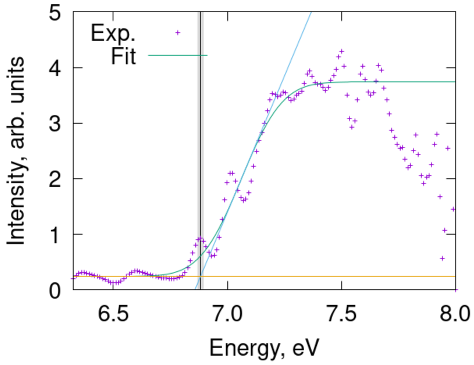

(e) $(\mathrm{Hel})_5$

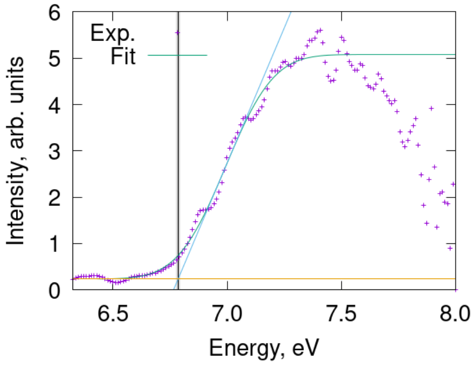

(f) $(\mathrm{Hel})_6$

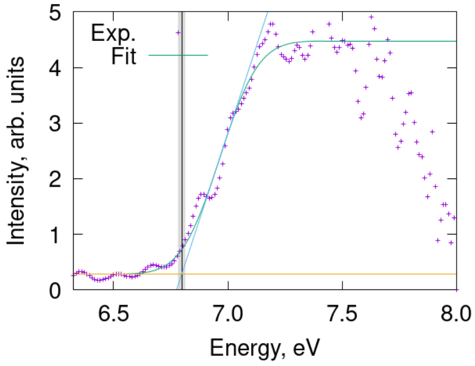

(g) $(\mathrm{Hel})_7$

Supplementary Figure 5: **Fitting results for the ionization thresholds of [4]helicene clusters** ($(\mathrm{Hel})_n$) as described above.

Supplementary Table 8: **Ionization thresholds** of [4]helicene clusters ($(\mathrm{Hel})_n$).

Cluster	Ionization threshold, eV
Monomer ($(\mathrm{Hel})_1$)	7.263 ± 0.007
Dimer ($(\mathrm{Hel})_2$)	7.163 ± 0.003
Trimer ($(\mathrm{Hel})_3$)	6.941 ± 0.007
Tetramer ($(\mathrm{Hel})_4$)	6.864 ± 0.023
Pentamer ($(\mathrm{Hel})_5$)	6.882 ± 0.014
Hexamer ($(\mathrm{Hel})_6$)	6.785 ± 0.009
Heptamer ($(\mathrm{Hel})_6$)	6.798 ± 0.016

Fit of the first peaks in PEPICO spectra of the [4]helicene monomer

The spectrum for the [4]helicene monomer in the range between 5.55 and 9.65 eV was fitted using five Gaussian functions in the form $I_0 \cdot \exp\left(-\frac{(x-x_0)^2}{w^2}\right)$ with paramters I_0 (peak height), x_0 (peak position), and w (peak width). The first two Gaussians represented the two-state peak around 7.7 eV, the third and fourth peaks represented the second two-state peak around 8.9 eV, and the last peak represented the baseline.

Supplementary Table 9: **Monomer spectral fit.** Results of the fit to the [4]helicene monomer spectrum, with x_0, w and I_0 being the peak position, width and height, respectively.

Peak number	x_0, eV	w, eV	I_0, arb. units
1	7.511 ± 0.002	0.130 ± 0.005	5.3 ± 0.2
2	7.783 ± 0.006	0.350 ± 0.007	8.5 ± 0.1
3	8.894 ± 0.002	0.120 ± 0.006	9 ± 1
4	9.051 ± 0.025	0.214 ± 0.020	5.9 ± 0.6
5	13 ± 3	3 ± 1	17 ± 26

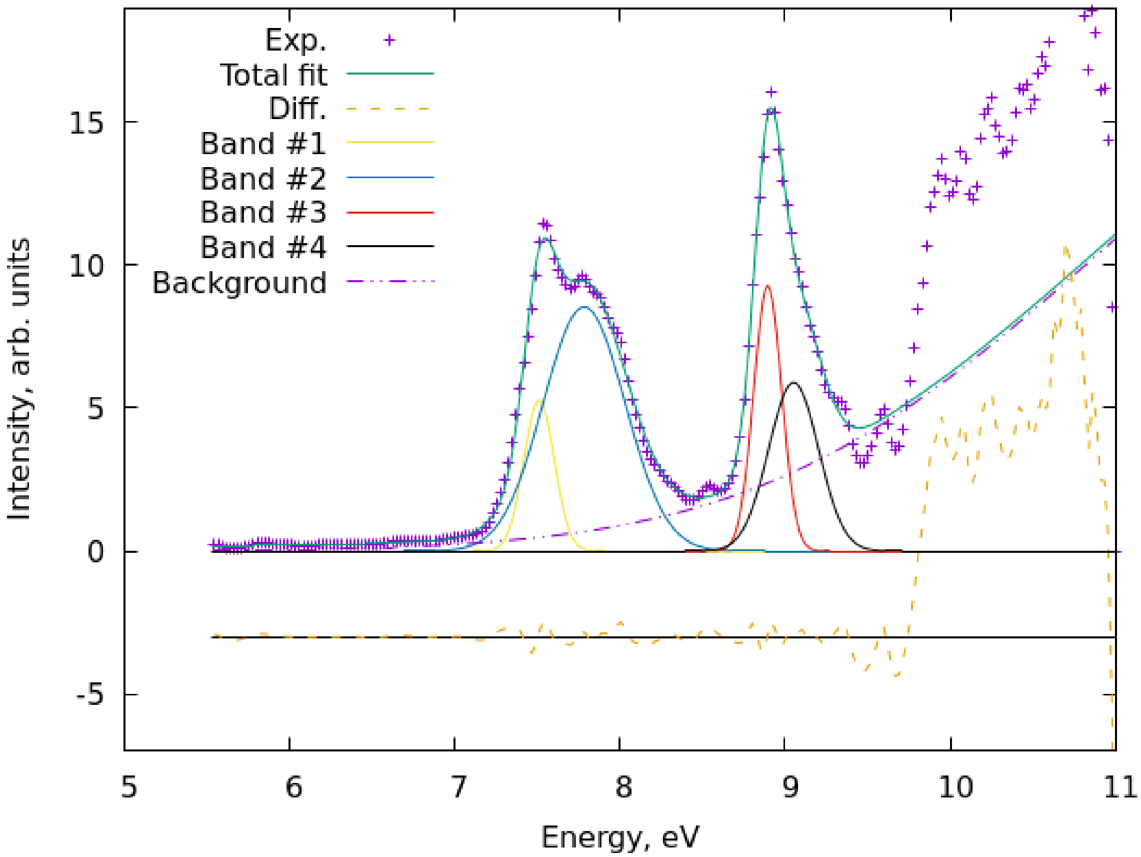

Supplementary Figure 6: **Fit of the [4]helicene monomer spectrum.**

Supplementary References

(1) Adamo, C.; Barone, V. Toward reliable density functional methods without adjustable parameters: The PBE0 model. *The Journal of Chemical Physics* **1999**, *110*, 6158–6170.

(2) Weigend, F.; Ahlrichs, R. Balanced basis sets of split valence, triple zeta valence and quadruple zeta valence quality for H to Rn: Design and assessment of accuracy. *Phys. Chem. Chem. Phys.* **2005**, *7*, 3297–3305.

(3) Gozem, S.; Krylov, A. I. The ezSpectra suite: An easy-to-use toolkit for spectroscopy modeling. *WIREs Computational Molecular Science* **2022**, e1546.

(4) Zhang, J.; Dolg, M. ABCluster: the artificial bee colony algorithm for cluster global optimization. *Phys. Chem. Chem. Phys.* **2015**, *17*, 24173–24181.

(5) Zhang, J.; Dolg, M. Global optimization of clusters of rigid molecules using the artificial bee colony algorithm. *Phys. Chem. Chem. Phys.* **2016**, *18*, 3003–3010.

(6) Zhang, J.; Glezakou, V.-A.; Rousseau, R.; Nguyen, M.-T. NWPEsSe: An Adaptive-Learning Global Optimization Algorithm for Nanosized Cluster Systems. *Journal of Chemical Theory and Computation* **2020**, *16*, 3947–3958, PMID: 32364725.

(7) Averkiev, B. B. Coalescence-Kick. `https://github.com/averkiev75/Coalescence-Kick`, 2013.

(8) Huang, W.; Sergeeva, A. P.; Zhai, H.-J.; Averkiev, B. B.; Wang, L.-S.; Boldyrev, A. I. A concentric planar doubly π-aromatic B19-cluster. *Nature Chemistry* **2010**, *2*, 202–206.

(9) Bannwarth, C.; Ehlert, S.; Grimme, S. GFN2-xTB—An Accurate and Broadly Parametrized Self-Consistent Tight-Binding Quantum Chemical Method with Multipole Electrostatics and Density-Dependent Dispersion Contributions. *Journal of Chemical Theory and Computation* **2019**, *15*, 1652–1671, PMID: 30741547.

(10) Bannwarth, C.; Caldeweyher, E.; Ehlert, S.; Hansen, A.; Pracht, P.; Seibert, J.; Spicher, S.; Grimme, S. Extended tight-binding quantum chemistry methods. *WIREs Computational Molecular Science* **2021**, *11*, e1493.

(11) Tikhonov, D. S. `https://stash.desy.de/projects/MOLINC`, 2020.

(12) Riplinger, C.; Neese, F. An efficient and near linear scaling pair natural orbital based local coupled cluster method. *J. Chem. Phys.* **2013**, *138*, 034106.

(13) Riplinger, C.; Sandhoefer, B.; Hansen, A.; Neese, F. Natural triple excitations in local coupled cluster calculations with pair natural orbitals. *J. Chem. Phys.* **2013**, *139*, 134101.

(14) Guo, Y.; Riplinger, C.; Becker, U.; Liakos, D. G.; Minenkov, Y.; Cavallo, L.; Neese, F. Communication: An improved linear scaling perturbative triples correction for the domain based local pair-natural orbital based singles and doubles coupled cluster method [DLPNO-CCSD(T)]. *J. Chem. Phys.* **2018**, *148*, 011101.

(15) Pal, S.; Rittby, M.; Bartlett, R. J.; Sinha, D.; Mukherjee, D. Multireference coupled-cluster methods using an incomplete model space: Application to ionization potentials and excitation energies of formaldehyde. *Chem. Phys. Lett.* **1987**, *137*, 273–278.

(16) Stanton, J. F.; Gauss, J. Analytic energy derivatives for ionized states described by the equation-of-motion coupled cluster method. *J. Chem. Phys.* **1994**, *101*, 8938–8944.

(17) A. I. Krylov, A. I. Equation-of-Motion Coupled-Cluster Methods for Open-Shell and Electronically Excited Species: The Hitchhiker's Guide to Fock Space. *Ann. Rev. Phys. Chem.* **2008**, *59*, 433–462.

(18) Tölle, J.; Deilmann, T.; Rohlfing, M.; Neugebauer, J. Subsystem-Based GW/Bethe–Salpeter Equation. *J. Chem. Theory Comput.* **2021**, *17*, 2186–2199.

(19) Pavanello, M.; Neugebauer, J. Modelling charge transfer reactions with the frozen density embedding formalism. *J. Chem. Phys.* **2011**, *135*, 234103.

(20) Pavanello, M.; Voorhis, T. V.; Visscher, L.; Neugebauer, J. An accurate and linear-scaling method for calculating charge-transfer excitation energies and diabatic couplings. *J. Chem. Phys.* **2013**, *138*, 054101.

(21) Solovyeva, A.; Pavanello, M.; Neugebauer, J. Describing Long-Range Charge-Separation Processes with Subsystem Density-Functional Theory. *J. Chem. Phys.* **2014**, *140*, 164103.

(22) Ramos, P.; Papadakis, M.; Pavanello, M. Performance of Frozen Density Embedding for Modeling Hole Transfer Reactions. *J. Phys. Chem. B* **2015**, *119*, 7541–7557.

(23) Neese, F. The ORCA program system. *Comput. Mol. Sci.* **2012**, *2*, 73–78.

(24) Neese, F. ORCA, an *ab initio*, density functional and SCF–MO package, Universität Bonn. http://www.thch.uni-bonn.de/tc/orca, 2009.

(25) Dunning, T. H. Gaussian basis sets for use in correlated molecular calculations. I. The atoms boron through neon and hydrogen. *J. Chem. Phys.* **1989**, *90*, 1007–1023.

(26) Weigend, F.; Köhn, A.; Hättig, C. Efficient use of the correlation consistent basis sets in resolution of the identity MP2 calculations. *J. Chem. Phys.* **2002**, *116*, 3175–3183.

(27) Schäfer, A.; Horn, H.; Ahlrichs, R. Fully optimized contracted Gaussian basis sets for atoms Li to Kr. *J. Chem. Phys.* **1992**, *97*, 2571–2577.

(28) Schäfer, A.; Huber, C.; Ahlrichs, R. Fully optimized contracted Gaussian basis sets of triple zeta valence quality for atoms Li to Kr. *J. Chem. Phys.* **1994**, *100*, 5829.

(29) Neese, F.; Wennmohs, F.; Hansen, A.; Becker, U. Efficient, approximate and parallel Hartree–Fock and hybrid DFT calculations. A 'chain-of-spheres' algorithm for the Hartree–Fock exchange. *Chem. Phys.* **2009**, *356*, 98–109.

(30) Unsleber, J. P.; Dresselhaus, T.; Klahr, K.; Schnieders, D.; Böckers, M.; Barton, D.; Neugebauer, J. SERENITY: A subsystem quantum chemistry program. *J. Comput. Chem.* **2018**, *39*, 788–798.

(31) Barton, D.; Bensberg, M.; Böckers, M.; Dresselhaus, T.; Eschenbach, P.; Hellmann, L.; Klahr, K.; Massolle, A.; Niemeyer, N.; Schnieders, D.; Tölle, J.; Unsleber, J. P.; Neugebauer, J. qcserenity/serenity: Release 1.3.1. **2020**, DOI: 10.5281/zenodo.4059510.

(32) Perdew, J. P.; Burke, K.; Ernzerhof, M. Generalized Gradient Approximation Made Simple. *Phys. Rev. Lett.* **1996**, *77*, 3865–3868.

(33) Lee, C.; Yang, W.; Parr, R. G. Development of the Colle-Salvetti correlation-energy formula into a functional of the electron density. *Phys. Rev. B* **1988**, *37*, 785–789.

(34) Becke, A. D. A new mixing of Hartree–Fock and local density-functional theories. *J. Chem. Phys.* **1993**, *98*, 1372–1377.

(35) Perdew, J. P.; Chevary, J. A.; Vosko, S. H.; Jackson, K. A.; Pederson, M. R.; Singh, D. J.; Fiolhais, C. Atoms, molecules, solids, and surfaces: Applications of the generalized gradient approximation for exchange and correlation. *Phys. Rev. B* **1992**, *46*, 6671.

(36) Perdew, J.; Wang, Y. *Electronic Structure of Solids'91*; Academie: Berlin, 1991; p 11.

(37) Lee, H.; Lee, C.; Parr, R. G. Conjoint gradient correction to the Hartree-Fock kinetic- and exchange-energy density functionals. *Phys. Rev. A* **1991**, *44*, 768–771.

(38) Lembarki, A.; Chermette, H. Obtaining a gradient-corrected kinetic-energy functional from the Perdew-Wang exchange functional. *Phys. Rev. A* **1994**, *50*, 5328–5331.

(39) Wesołowski, T. A.; Weber, J. Kohn–Sham equations with constrained electron density: An iterative evaluation of the ground-state electron density of interacting molecules. *Chem. Phys. Lett.* **1996**, *248*, 71–76.

(40) Artiukhin, D. G.; Neugebauer, J. Frozen-density embedding as a quasi-diabatization tool: Charge-localized states for spin-density calculations. *J. Chem. Phys.* **2018**, *148*, 214104.

(41) Artiukhin, D. G.; Eschenbach, P.; Neugebauer, J. Computational Investigation of the Spin-Density Asymmetry in Photosynthetic Reaction Center Models from First Principles. *J. Phys. Chem. B* **2020**, *124*, 4873–4888.

(42) Artiukhin, D. G.; Eschenbach, P.; Matysik, J.; Neugebauer, J. Theoretical Assessment of Hinge-Type Models for Electron Donors in Reaction Centers of Photosystems I and II as Well as of Purple Bacteria. *J. Phys. Chem. B* **2021**, *125*, 3066–3079.

(43) Duchemin, I.; Blase, X. Robust Analytic-Continuation Approach to Many-Body GW Calculations. *J. Chem. Theory Comput.* **2020**, *16*, 1742–1756.

(44) Vidberg, H. J.; Serene, J. W. Solving the Eliashberg equations by means of N-point Padé approximants. *J. Low Temp. Phys.* **1977**, *29*, 179–192.

(45) van Setten, M. J.; Caruso, F.; Sharifzadeh, S.; Ren, X.; Scheffler, M.; Liu, F.; Lischner, J.; Lin, L.; Deslippe, J. R.; Louie, S. G.; Yang, C.; Weigend, F.; Neaton, J. B.; Evers, F.; Rinke, P. GW100: Benchmarking G0W0 for Molecular Systems. *J. Chem. Theory Comput.* **2015**, *11*, 5665–5687.

(46) Dontot, L.; Suaud, N.; Rapacioli, M.; Spiegelman, F. An extended DFTB-CI model for charge-transfer excited states in cationic molecular clusters: model studies versus ab initio calculations in small PAH clusters. *Phys. Chem. Chem. Phys.* **2016**, *18*, 3545–3557.

(47) Joblin, C.; Dontot, L.; Garcia, G. A.; Spiegelman, F.; Rapacioli, M.; Nahon, L.; Parneix, P.; Pino, T.; Bréchignac, P. Size Effect in the Ionization Energy of PAH Clusters. *The Journal of Physical Chemistry Letters* **2017**, *8*, 3697–3702, PMID: 28742357.

(48) Koster, A. et al. deMon2k. 2018.

(49) Ásgeirsson, V.; Birgisson, B. O.; Bjornsson, R.; Becker, U.; Neese, F.; Riplinger, C.; Jónsson, H. Nudged Elastic Band Method for Molecular Reactions Using Energy-Weighted Springs Combined with Eigenvector Following. *Journal of Chemical Theory and Computation* **2021**, *17*, 4929–4945, PMID: 34275279.

(50) Rosenblatt, M. Remarks on Some Nonparametric Estimates of a Density Function. *Ann. MAth. Statist.* **1956**, *27*, 832 – 837.

(51) Parzen, E. On Estimation of a Probability Density Function and Mode. *Ann. MAth. Statist.* **1962**, *33*, 1065 – 1076.

(52) Hunter, J. D. Matplotlib: A 2D graphics environment. *Comput. Sci. Eng.* **2007**, *9*, 90–95.

(53) Waskom, M. L. Seaborn: statistical data visualization. *J. Open Source Softw.* **2021**, *6*, 3021.
