# Supplementary material for: Evolution of the ionisation energy with the stepwise growth of chiral clusters of [4]helicene
Source: Nat Commun. 2024 Jun 10;15:4928. doi: 10.1038/s41467-024-48778-0 (PMC11164862; doi:10.1038/s41467-024-48778-0)
Supplement: Supplementary file 3 — Description of Additional Supplementary Files [file 41467_2024_48778_MOESM3_ESM.pdf]

Description of Additional Supplementary Files:

Supplementary Data 1: We provide cartesian coordinates and molecular structures for all the [4]helicene clusters (up to the heptamer and below 210 cm-1 in energy with respect to the lowest energy form for each cluster size) computed at the GFN2-xTB level of theory. The structures and filenames indicate the enantiomeric combinations of each cluster, using the same naming rules as used in the main article and supplementary information.
