# Supplementary figures and images for: Evolution of the ionisation energy with the stepwise growth of chiral clusters of [4]helicene
Source: Nat Commun. 2024 Jun 10;15:4928. doi: 10.1038/s41467-024-48778-0 (PMC11164862; doi:10.1038/s41467-024-48778-0)

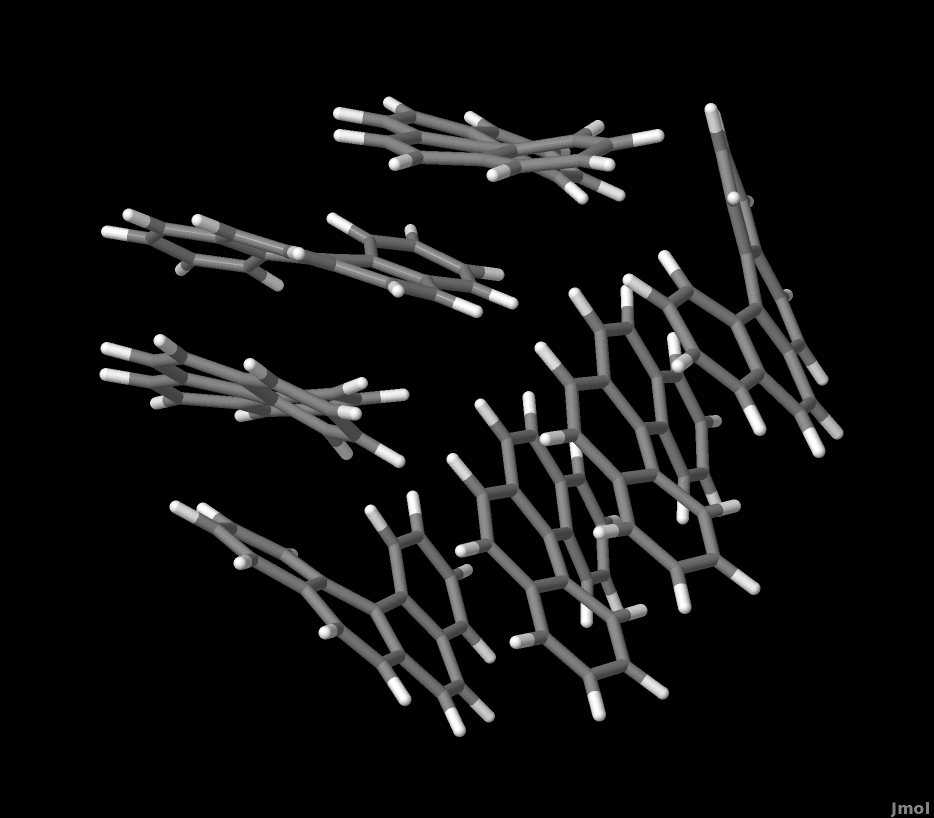

Supplement: Supplementary file 4 — Supplementary Data 1 [file 41467_2024_48778_MOESM4_ESM.zip › StructuresBelow210wvnThr/PPPPPPM.jpg]

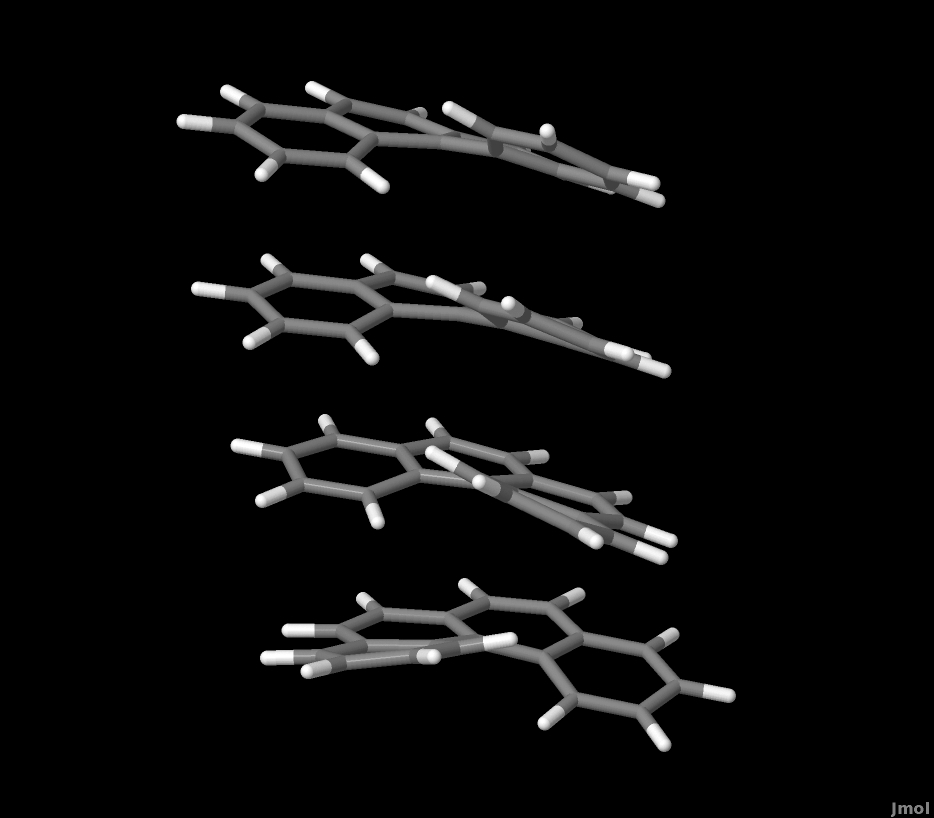

Supplement: Supplementary file 4 — Supplementary Data 1 [file 41467_2024_48778_MOESM4_ESM.zip › StructuresBelow210wvnThr/PPPM.jpg]

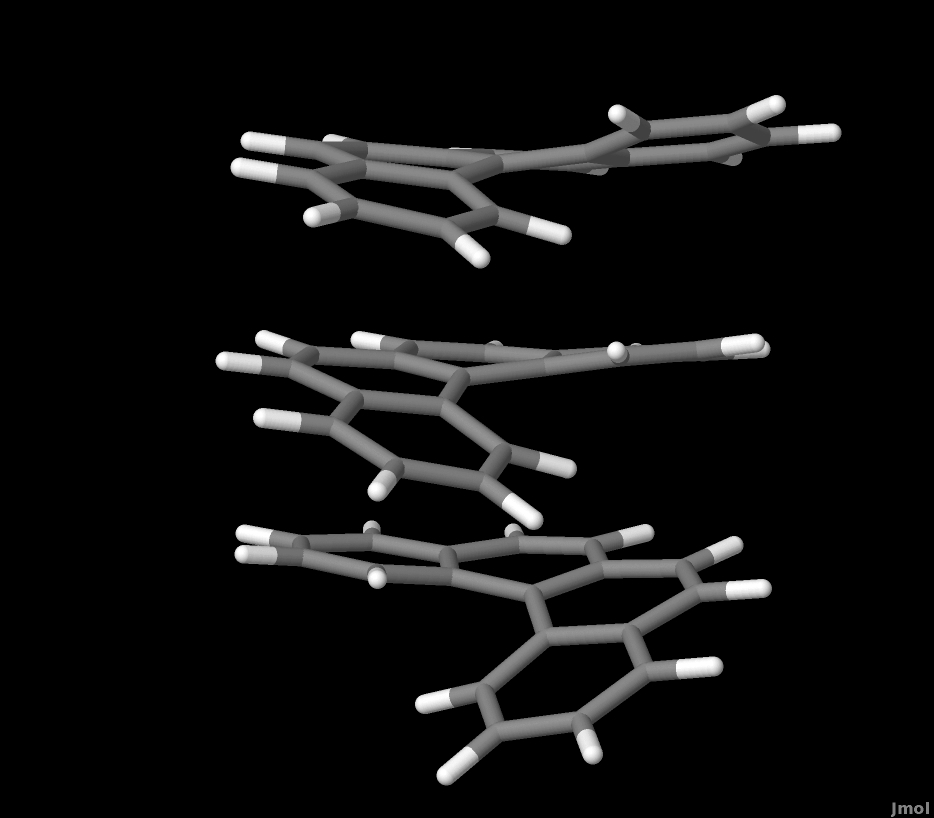

Supplement: Supplementary file 4 — Supplementary Data 1 [file 41467_2024_48778_MOESM4_ESM.zip › StructuresBelow210wvnThr/PPM.jpg]

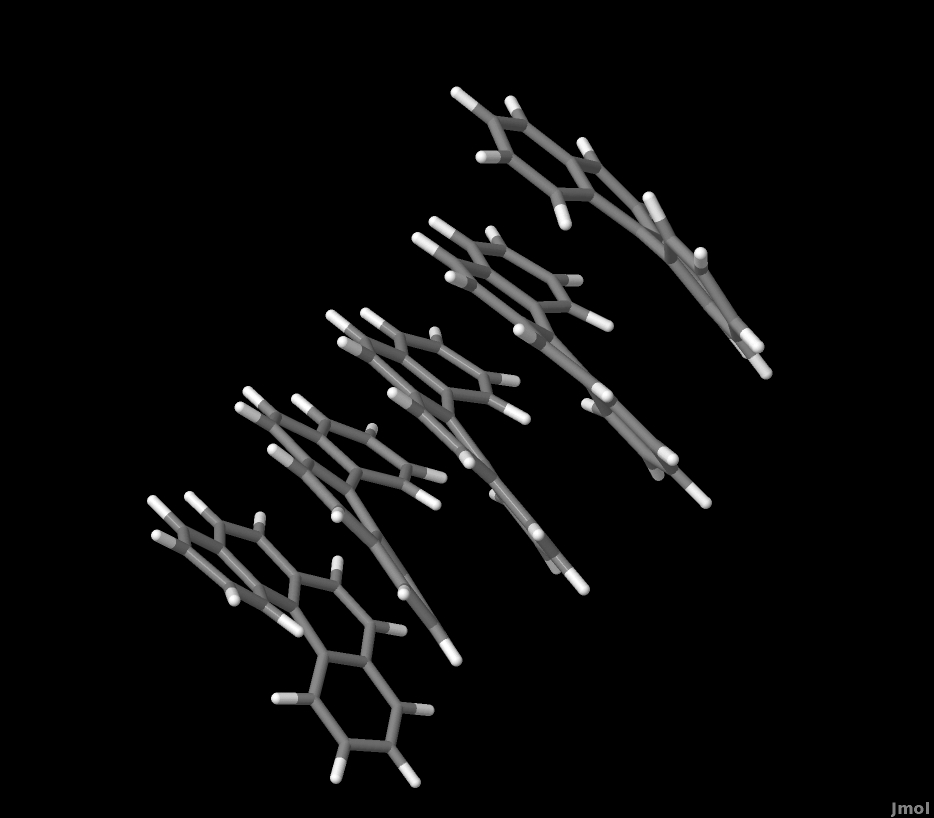

Supplement: Supplementary file 4 — Supplementary Data 1 [file 41467_2024_48778_MOESM4_ESM.zip › StructuresBelow210wvnThr/PPPPM.jpg]

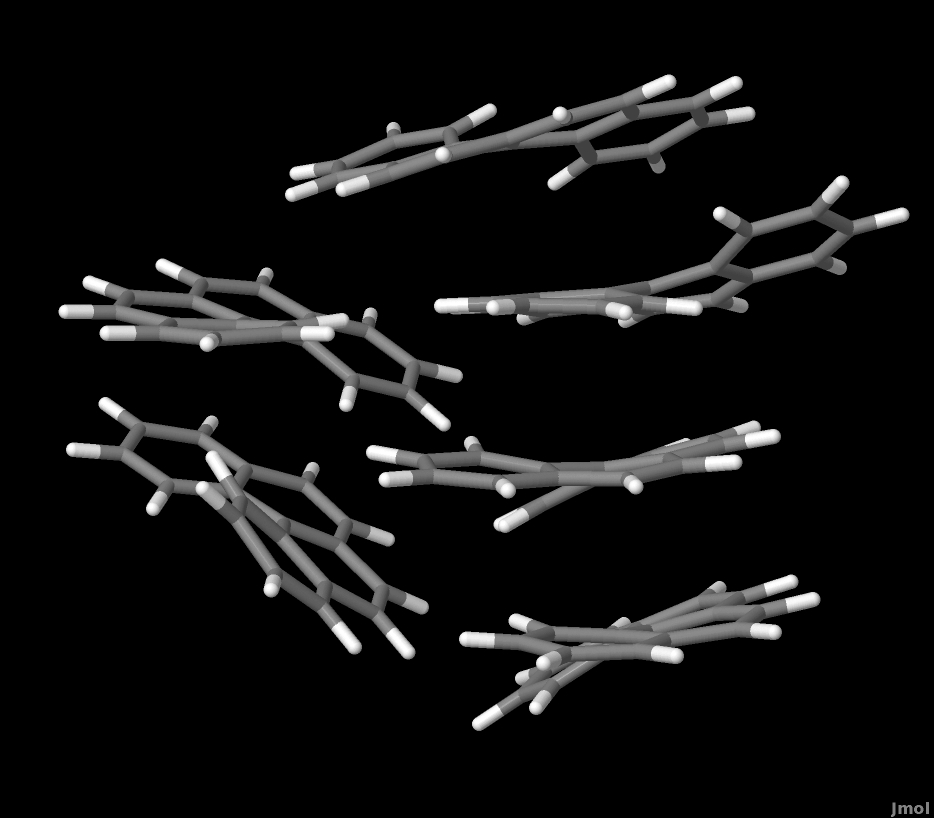

Supplement: Supplementary file 4 — Supplementary Data 1 [file 41467_2024_48778_MOESM4_ESM.zip › StructuresBelow210wvnThr/PPPPPM.jpg]

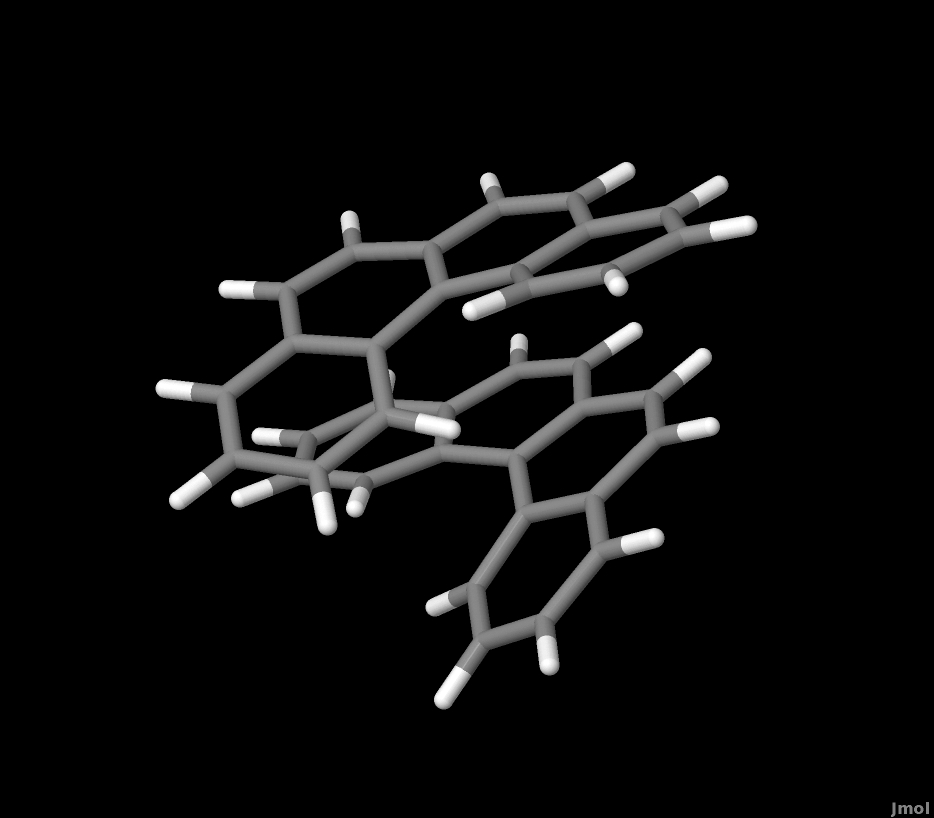

Supplement: Supplementary file 4 — Supplementary Data 1 [file 41467_2024_48778_MOESM4_ESM.zip › StructuresBelow210wvnThr/PM.jpg]

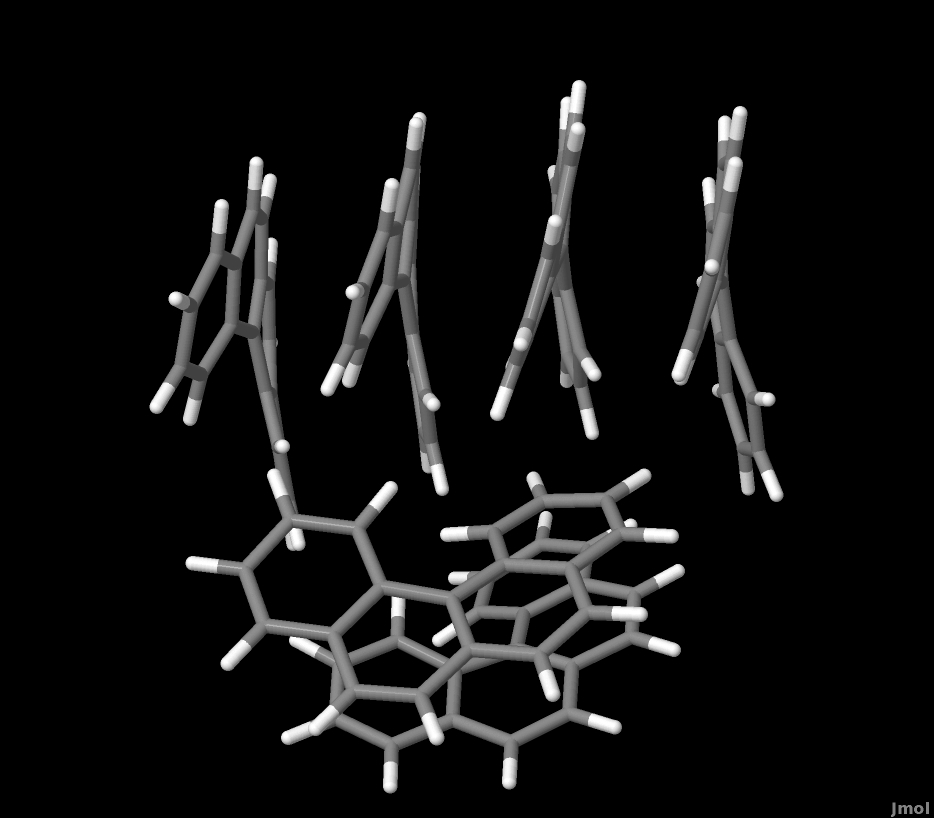

Supplement: Supplementary file 4 — Supplementary Data 1 [file 41467_2024_48778_MOESM4_ESM.zip › StructuresBelow210wvnThr/PPPPPP.jpg]

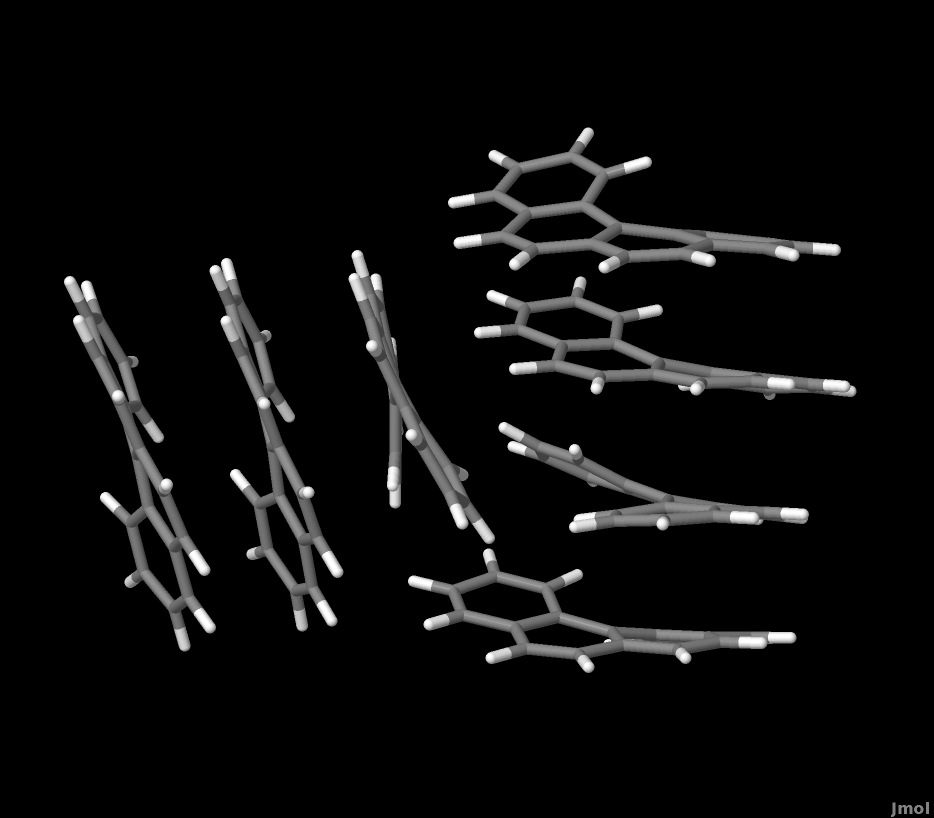

Supplement: Supplementary file 4 — Supplementary Data 1 [file 41467_2024_48778_MOESM4_ESM.zip › StructuresBelow210wvnThr/PPPPPMM.jpg]

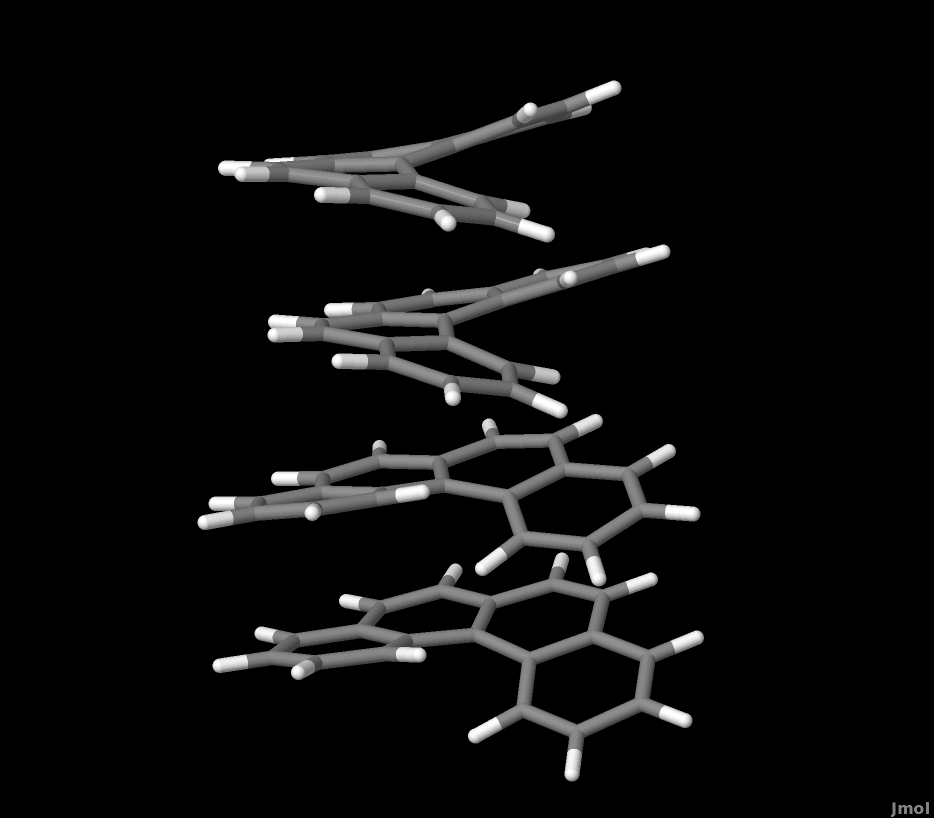

Supplement: Supplementary file 4 — Supplementary Data 1 [file 41467_2024_48778_MOESM4_ESM.zip › StructuresBelow210wvnThr/PPMM.jpg]

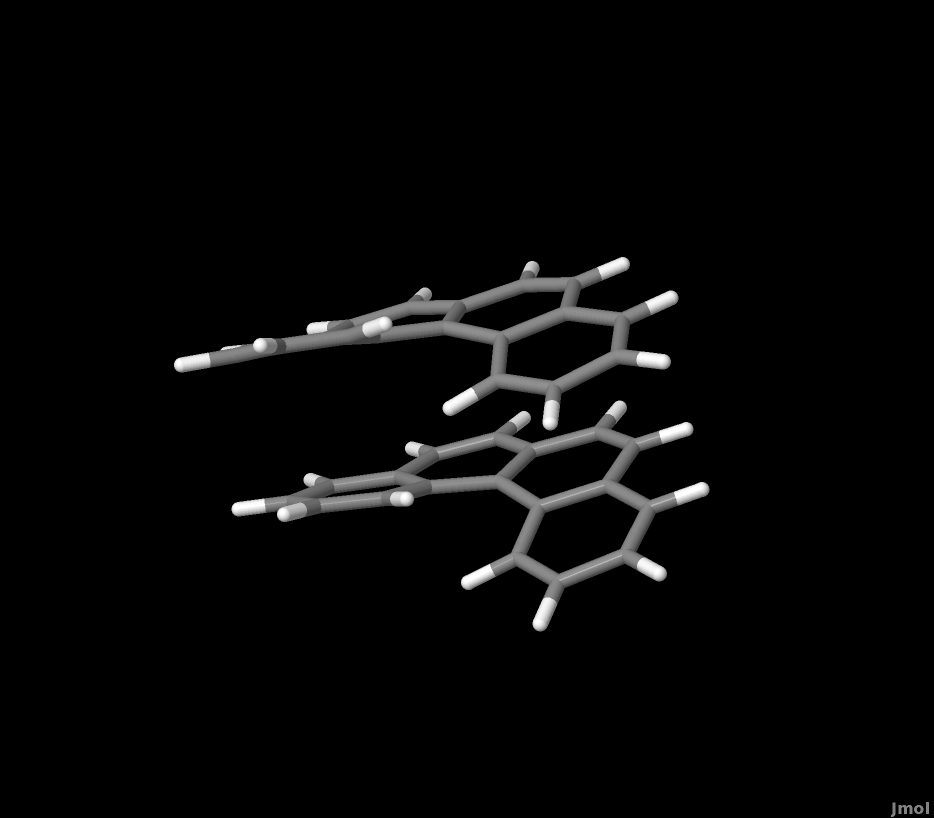

Supplement: Supplementary file 4 — Supplementary Data 1 [file 41467_2024_48778_MOESM4_ESM.zip › StructuresBelow210wvnThr/PP.jpg]

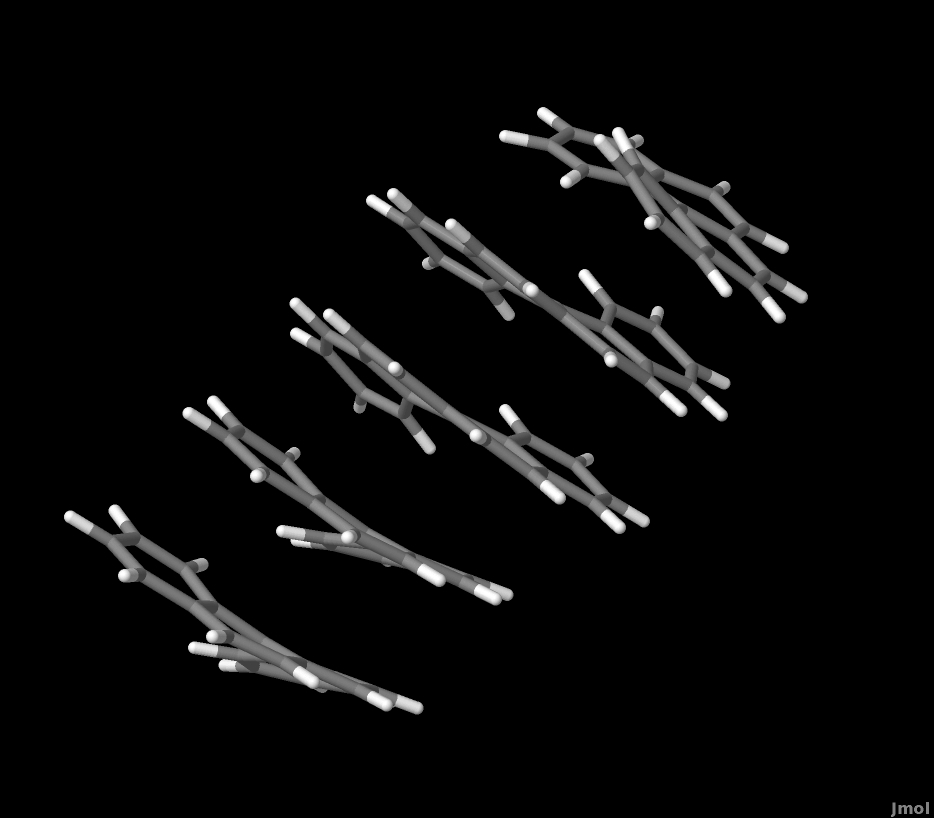

Supplement: Supplementary file 4 — Supplementary Data 1 [file 41467_2024_48778_MOESM4_ESM.zip › StructuresBelow210wvnThr/PPPMM.jpg]

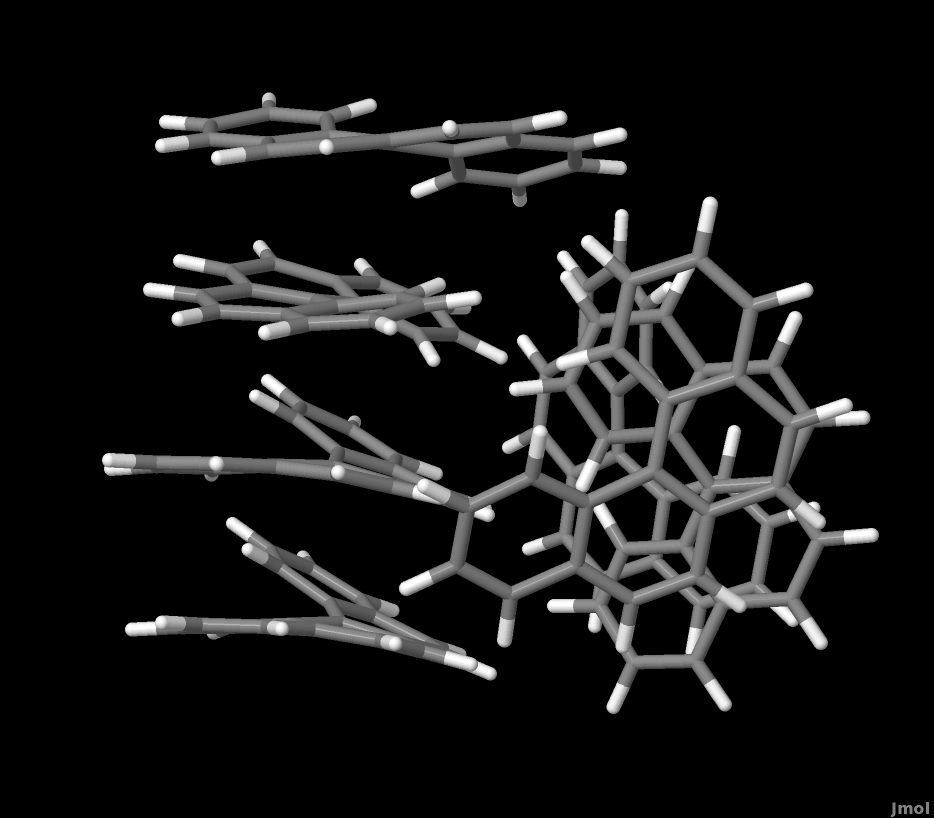

Supplement: Supplementary file 4 — Supplementary Data 1 [file 41467_2024_48778_MOESM4_ESM.zip › StructuresBelow210wvnThr/PPPPMMM.jpg]

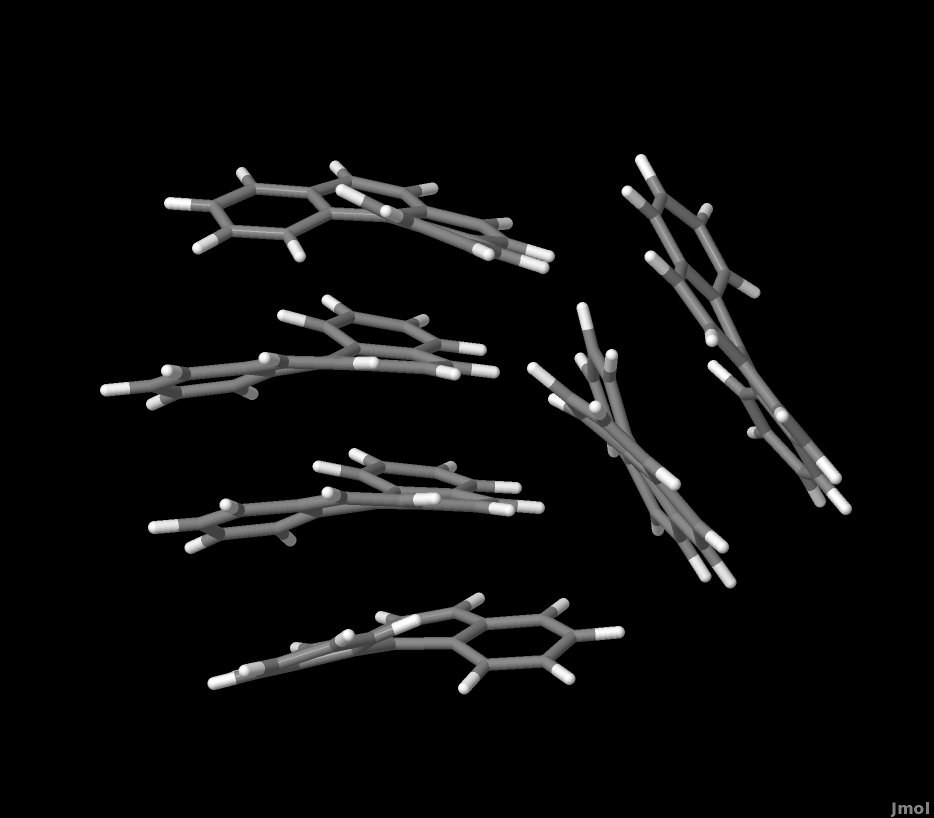

Supplement: Supplementary file 4 — Supplementary Data 1 [file 41467_2024_48778_MOESM4_ESM.zip › StructuresBelow210wvnThr/PPPPMM.jpg]

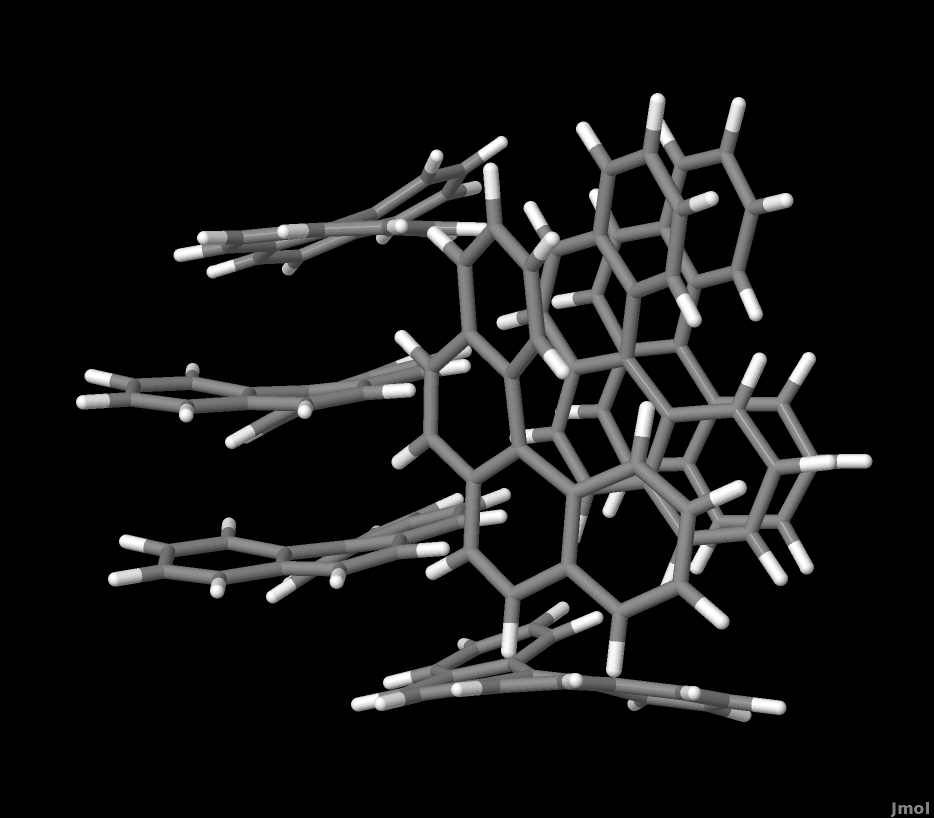

Supplement: Supplementary file 4 — Supplementary Data 1 [file 41467_2024_48778_MOESM4_ESM.zip › StructuresBelow210wvnThr/PPPPPPP.jpg]

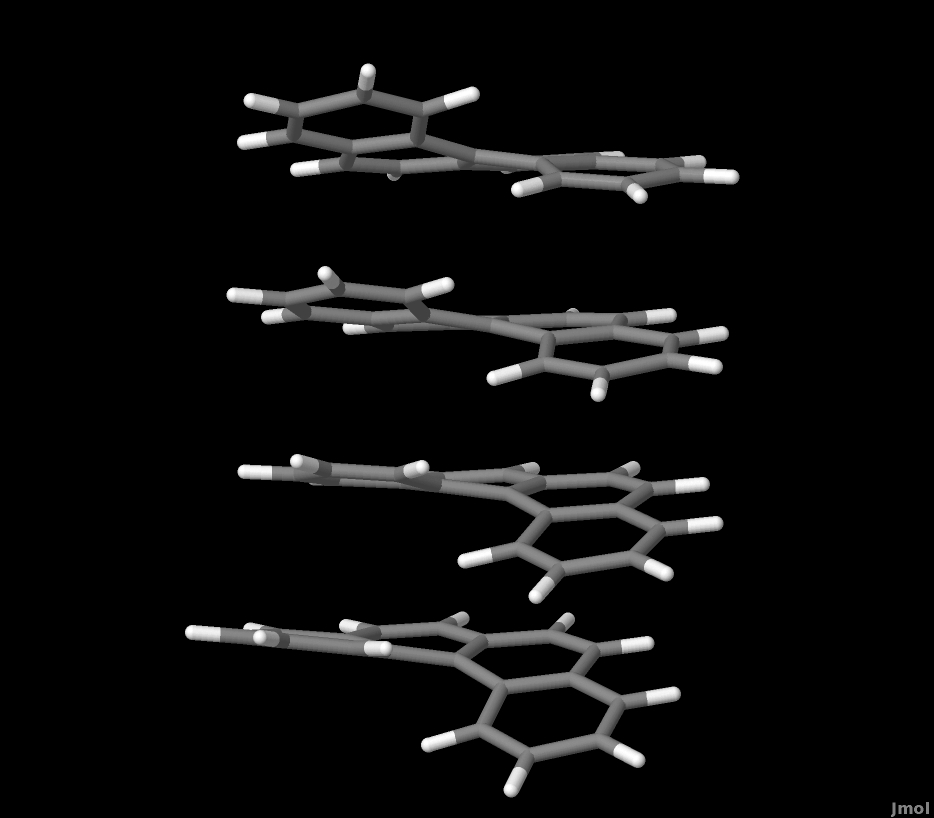

Supplement: Supplementary file 4 — Supplementary Data 1 [file 41467_2024_48778_MOESM4_ESM.zip › StructuresBelow210wvnThr/PPPP.jpg]

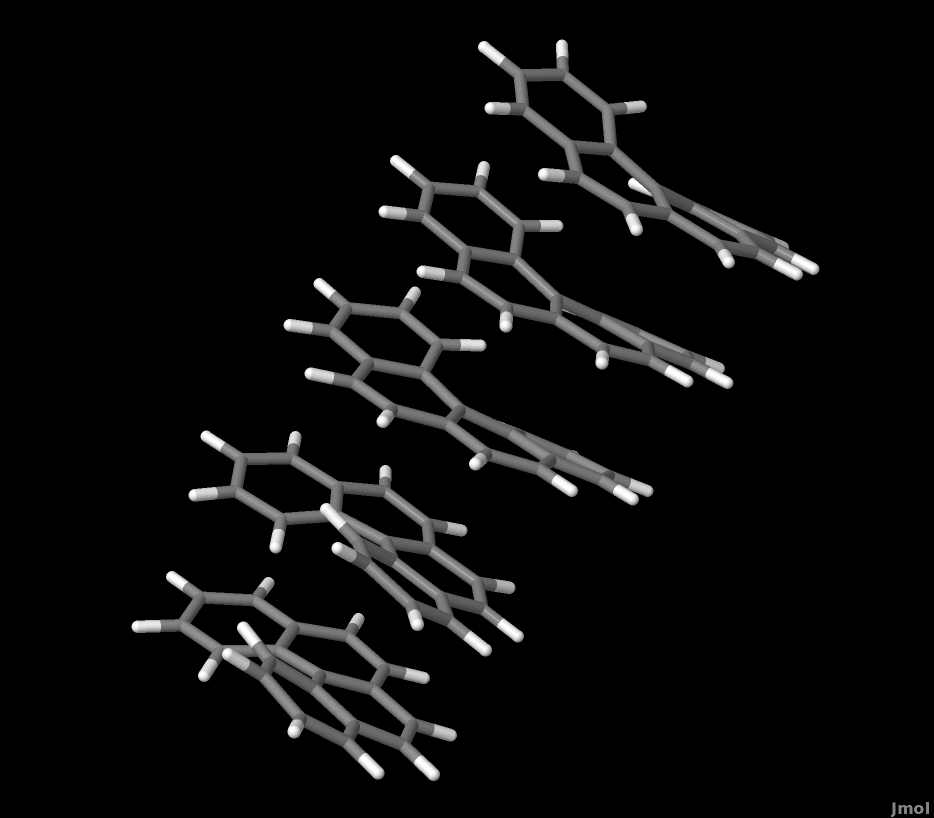

Supplement: Supplementary file 4 — Supplementary Data 1 [file 41467_2024_48778_MOESM4_ESM.zip › StructuresBelow210wvnThr/PPPPP.jpg]

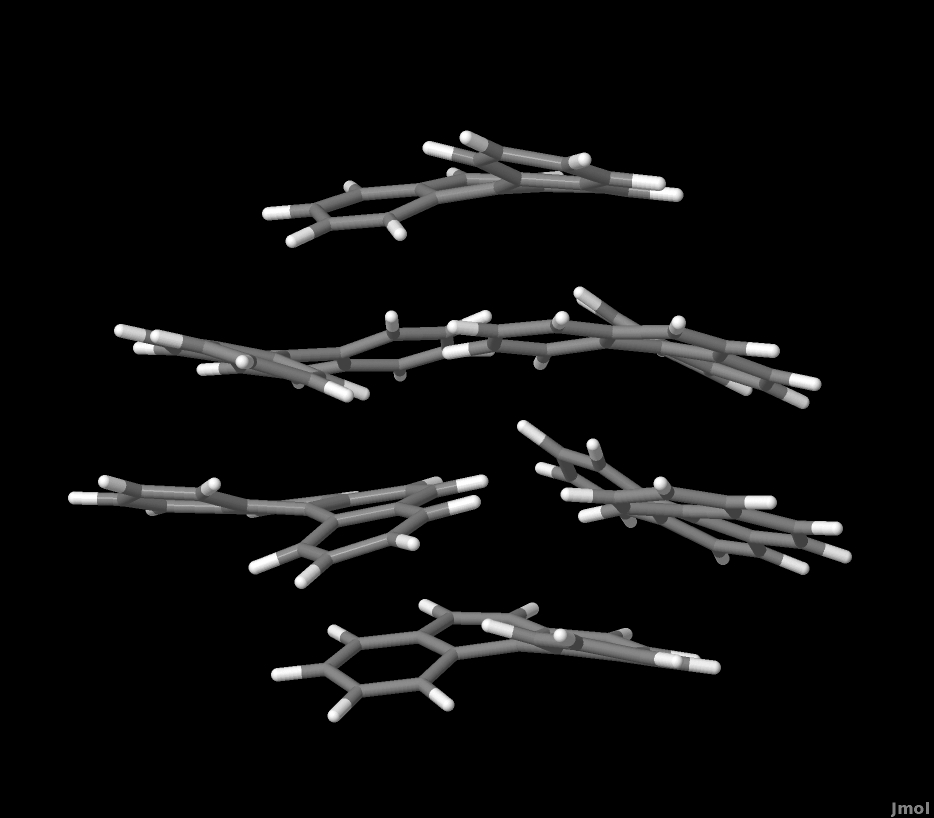

Supplement: Supplementary file 4 — Supplementary Data 1 [file 41467_2024_48778_MOESM4_ESM.zip › StructuresBelow210wvnThr/PPPMMM.jpg]

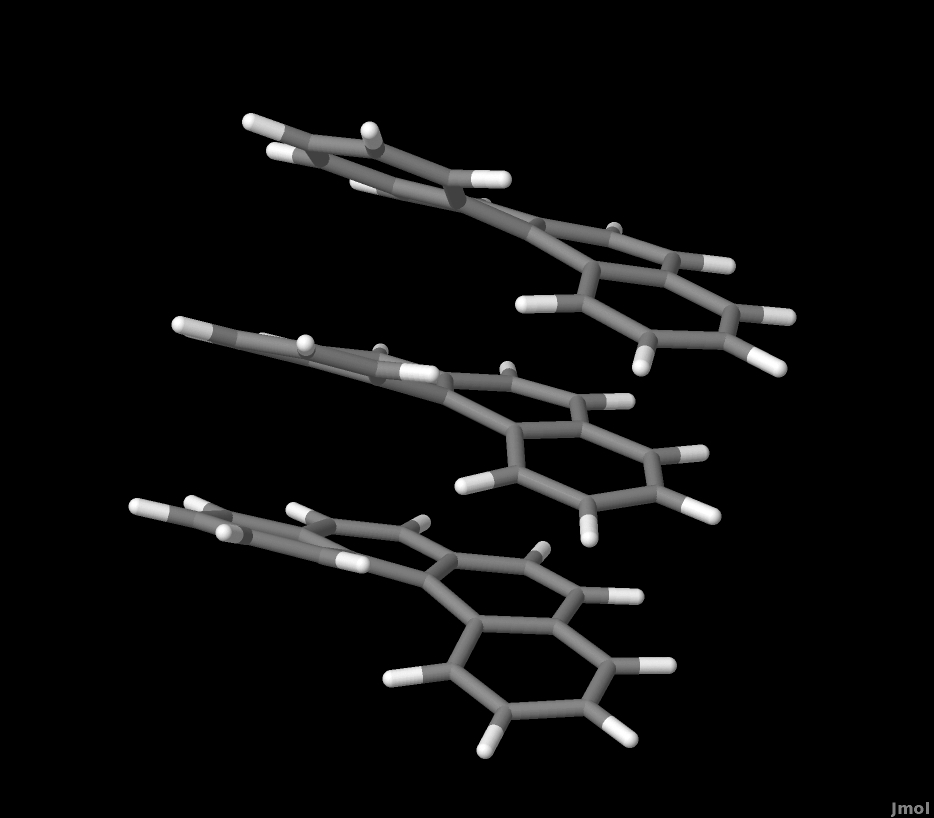

Supplement: Supplementary file 4 — Supplementary Data 1 [file 41467_2024_48778_MOESM4_ESM.zip › StructuresBelow210wvnThr/PPP.jpg]
